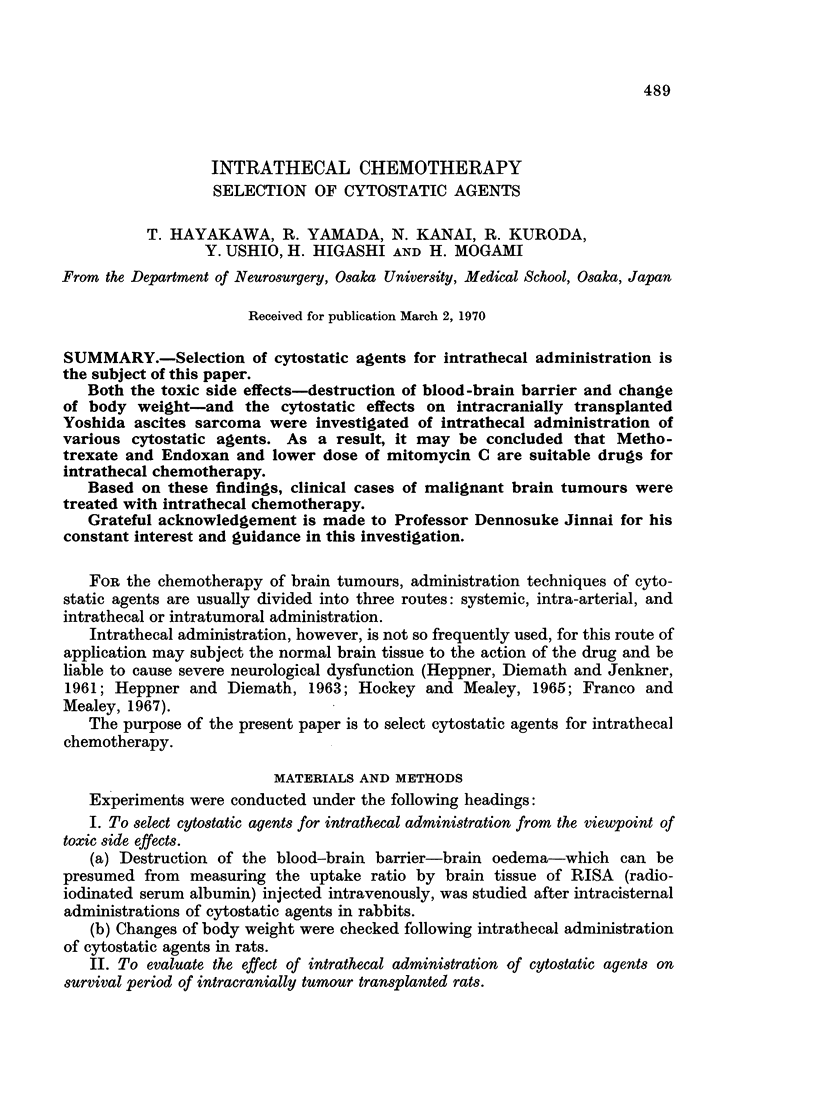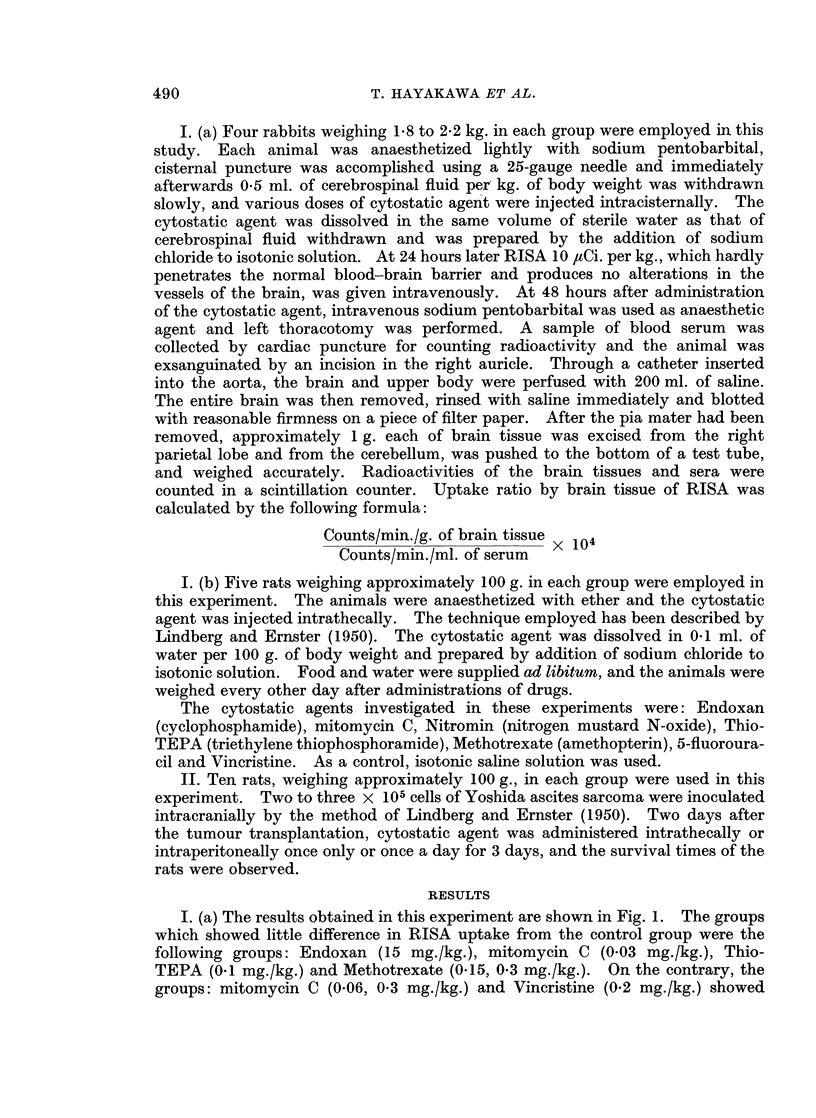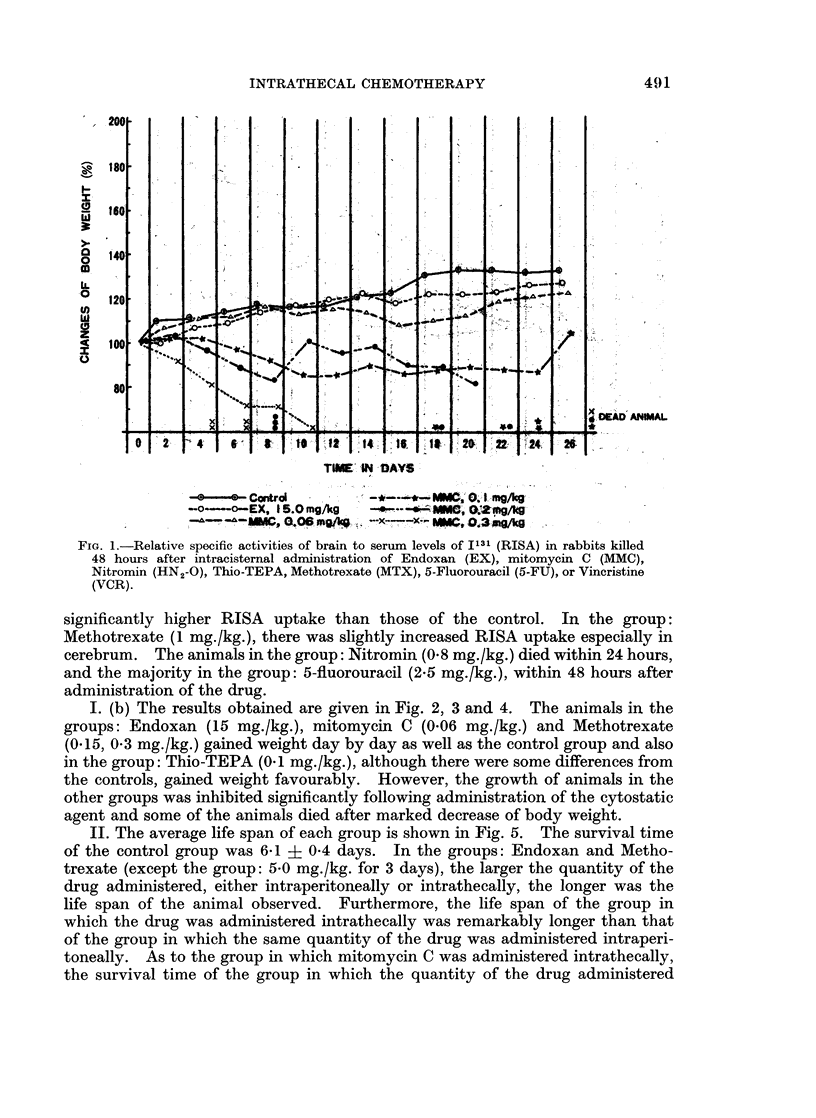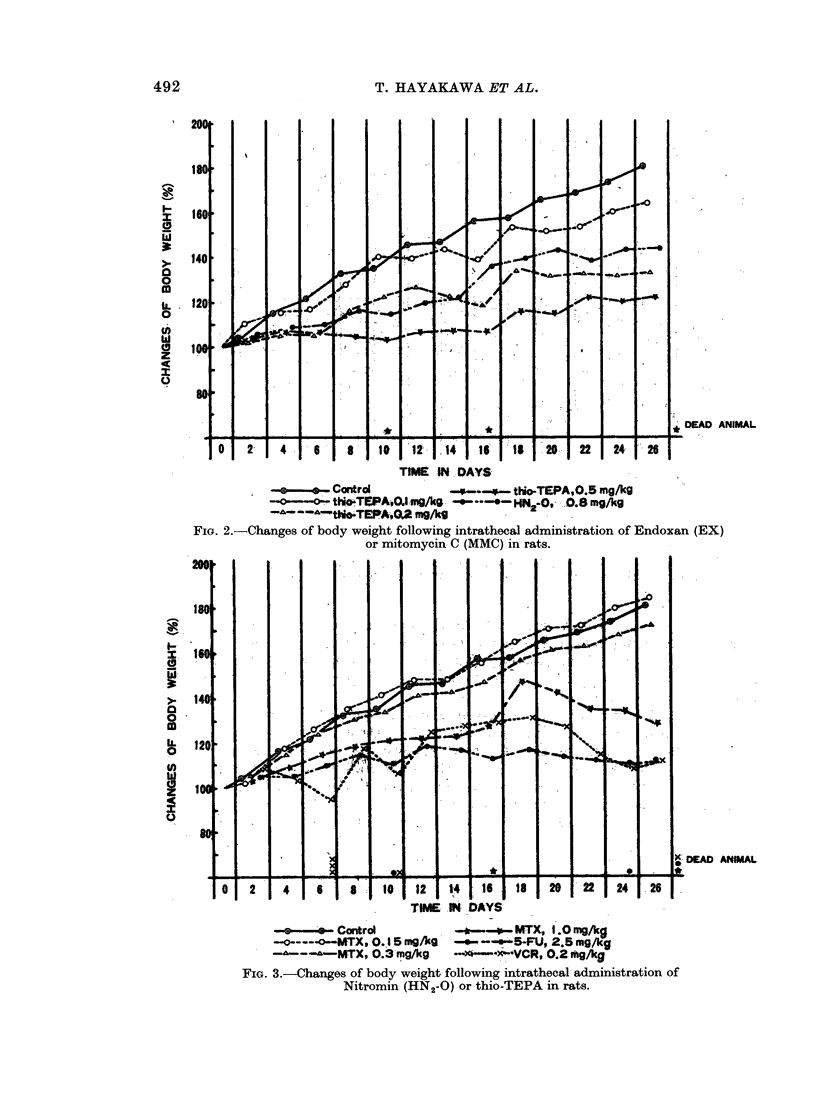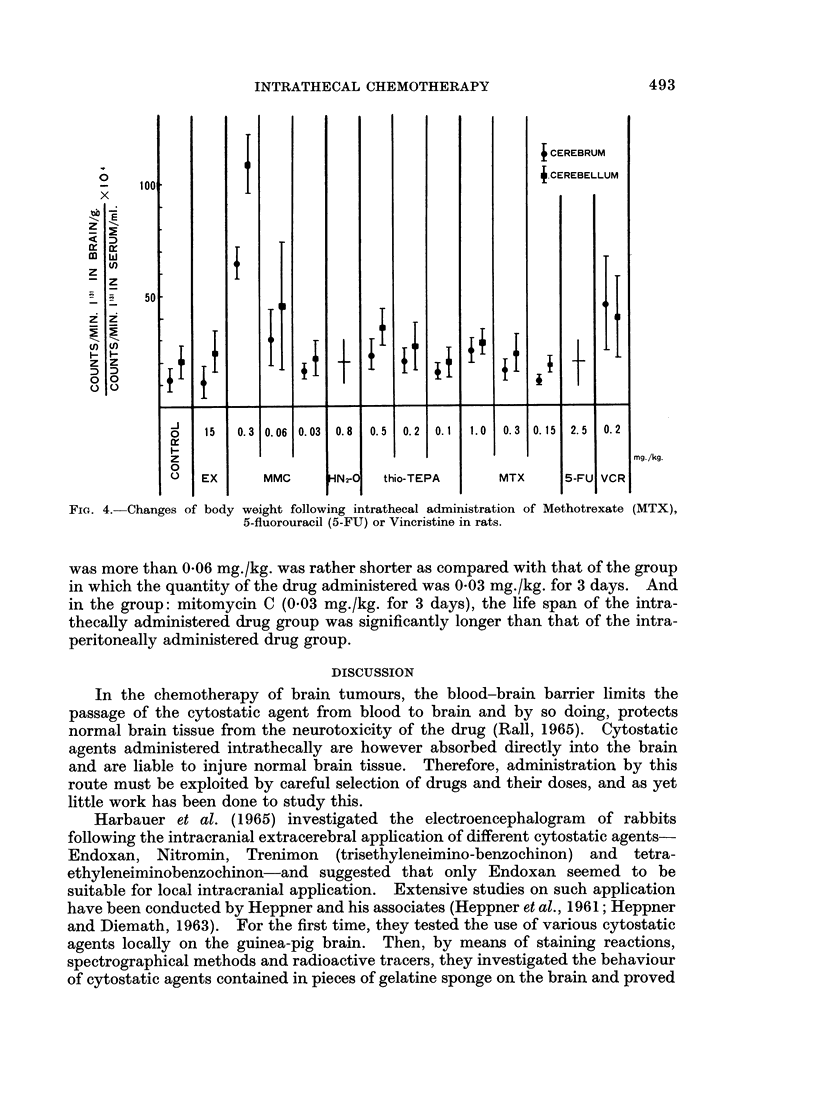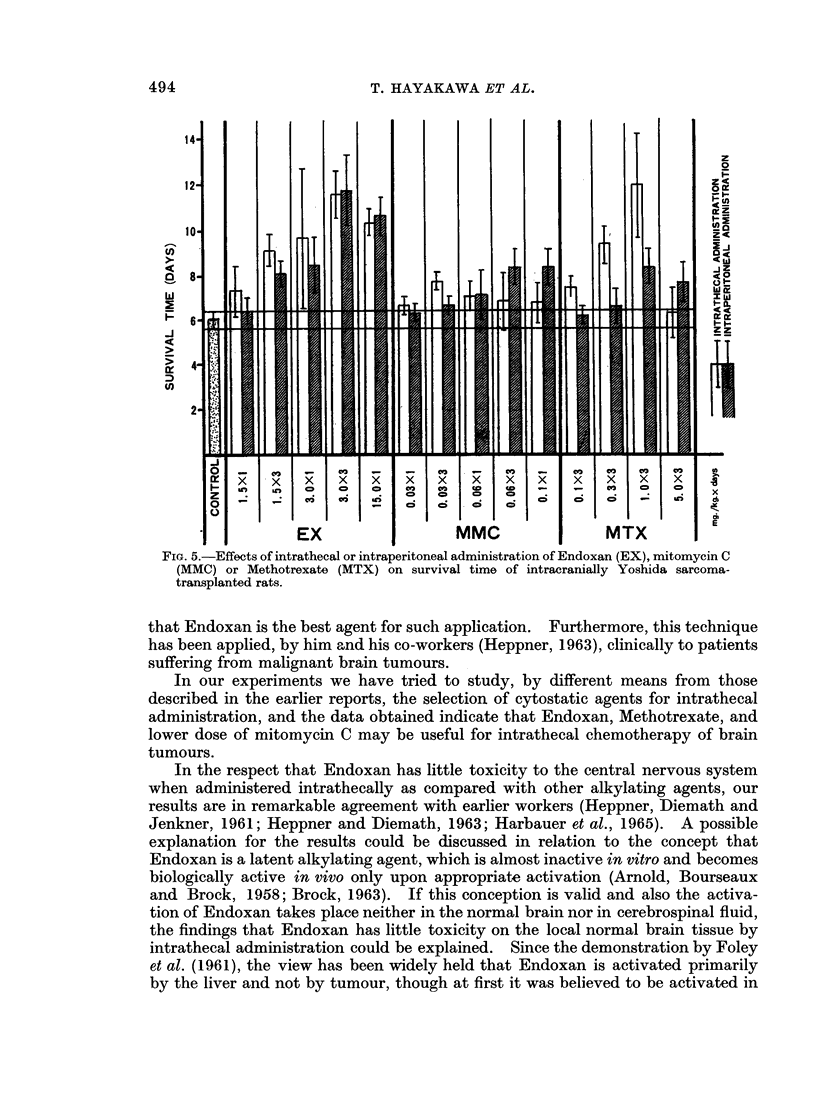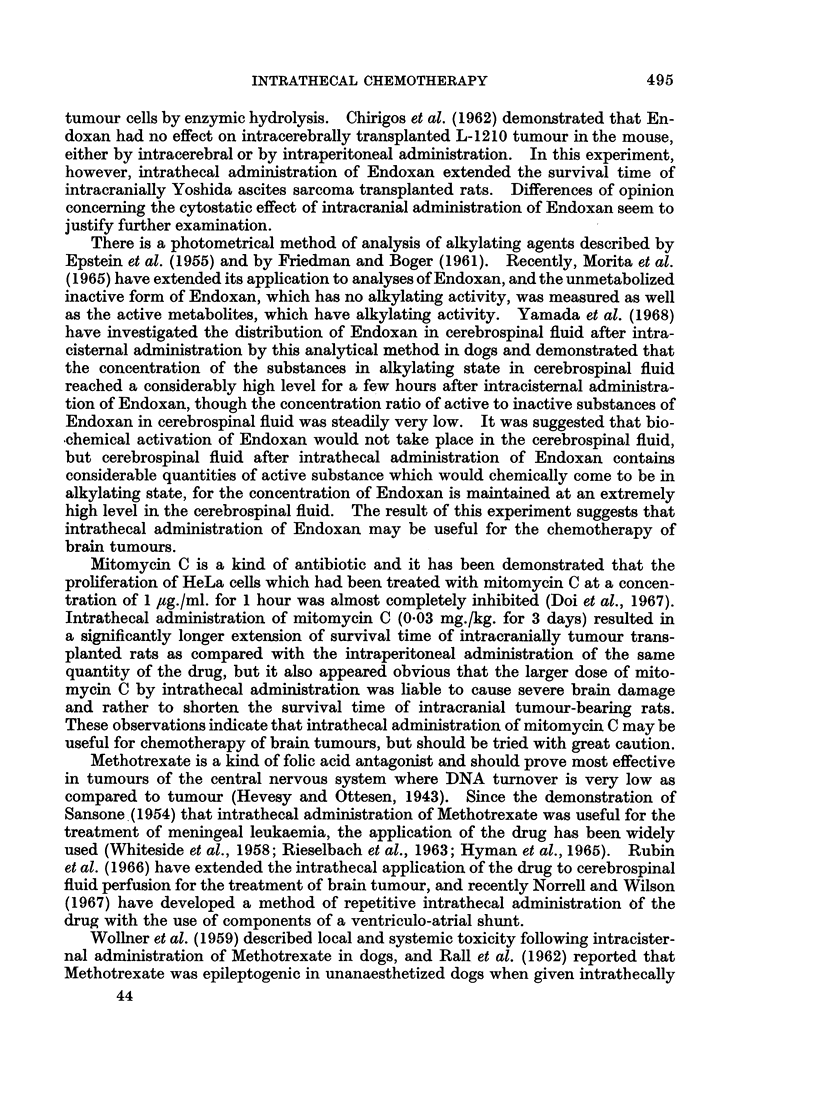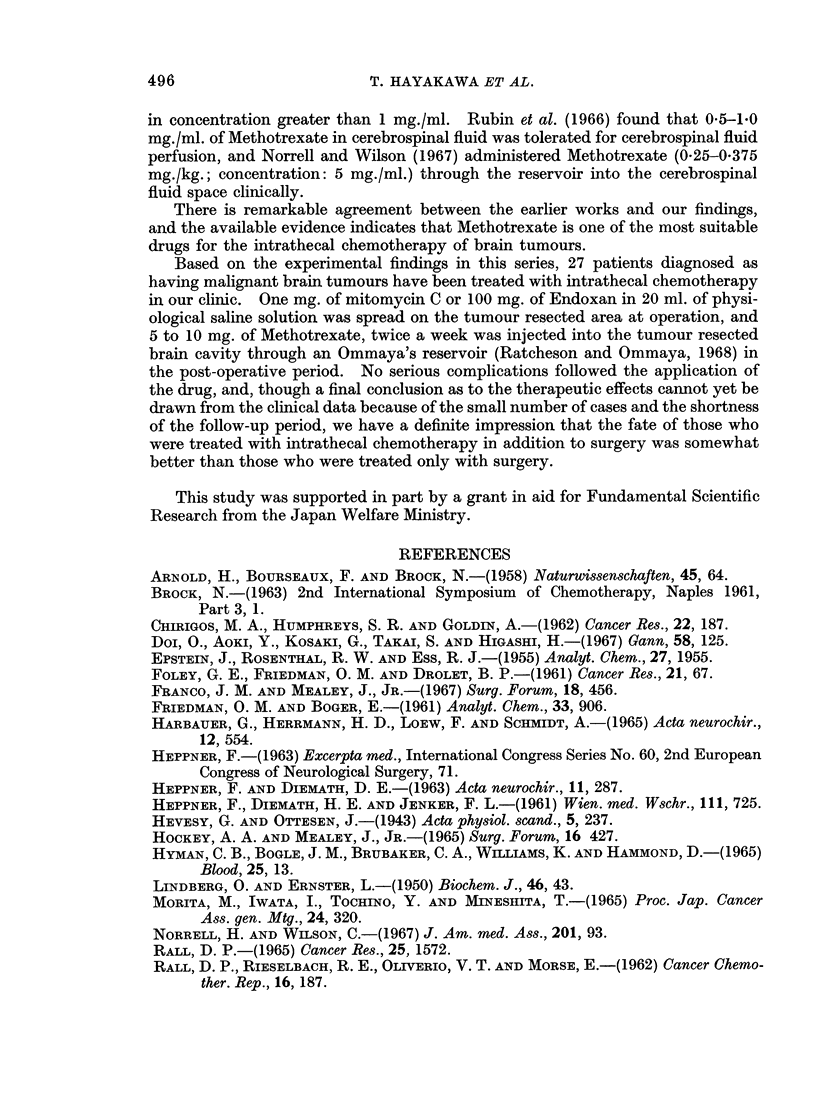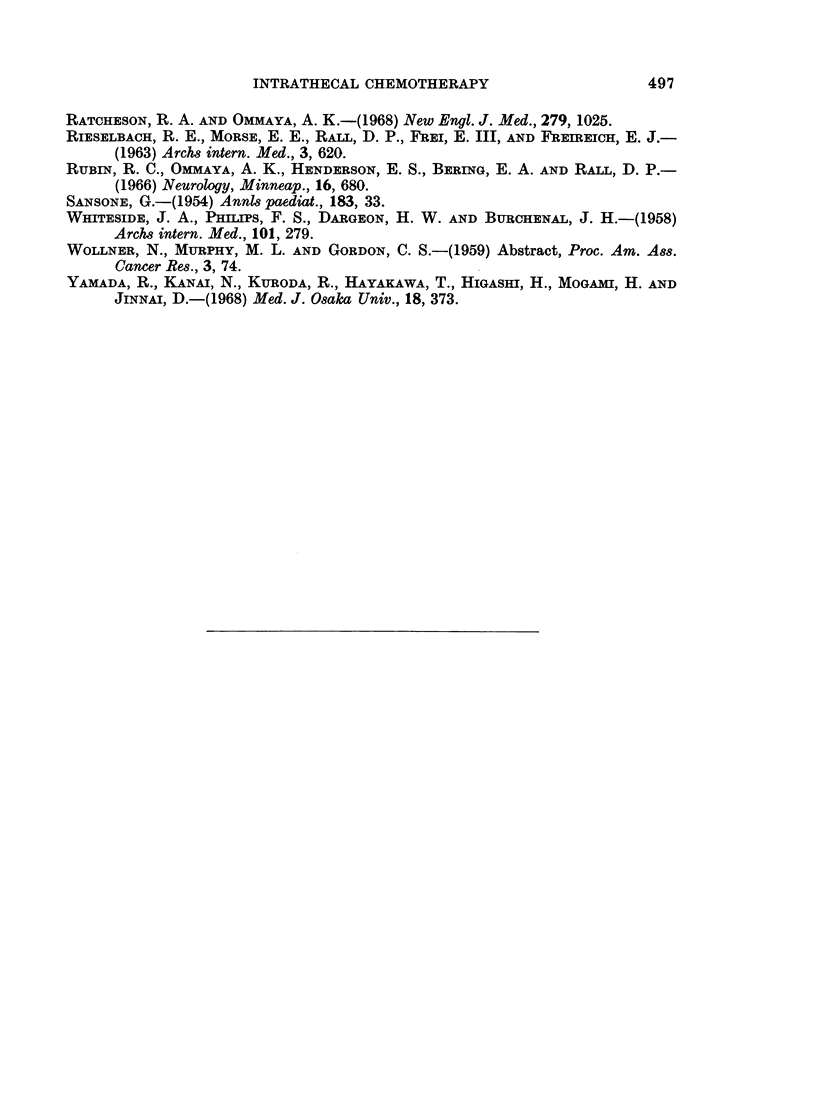# Intrathecal Chemotherapy

**DOI:** 10.1038/bjc.1970.59

**Published:** 1970-09

**Authors:** T. Hayakawa, R. Yamada, N. Kanai, R. Kuroda, Y. Ushio, H. Higashi, H. Mogami

## Abstract

Selection of cytostatic agents for intrathecal administration is the subject of this paper.

Both the toxic side effects—destruction of blood-brain barrier and change of body weight—and the cytostatic effects on intracranially transplanted Yoshida ascites sarcoma were investigated of intrathecal administration of various cytostatic agents. As a result, it may be concluded that Methotrexate and Endoxan and lower dose of mitomycin C are suitable drugs for intrathecal chemotherapy.

Based on these findings, clinical cases of malignant brain tumours were treated with intrathecal chemotherapy.

Grateful acknowledgement is made to Professor Dennosuke Jinnai for his constant interest and guidance in this investigation.


### INTRATHECAL CHEMOTHERAPY
### SELECTION OF CYTOSTATIC AGENTS

T. HAYAKAWA, R. YAMADA, N. KANAI, R. KURODA,
Y. USHIO, H. HIGASHI AND H. MOGAMI

*From the Department of Neurosurgery, Osaka University, Medical School, Osaka, Japan*

Received for publication March 2, 1970

**SUMMARY.—Selection of cytostatic agents for intrathecal administration is the subject of this paper.**

**Both the toxic side effects—destruction of blood-brain barrier and change of body weight—and the cytostatic effects on intracranially transplanted Yoshida ascites sarcoma were investigated of intrathecal administration of various cytostatic agents. As a result, it may be concluded that Methotrexate and Endoxan and lower dose of mitomycin C are suitable drugs for intrathecal chemotherapy.**

**Based on these findings, clinical cases of malignant brain tumours were treated with intrathecal chemotherapy.**

**Grateful acknowledgement is made to Professor Dennosuke Jinnai for his constant interest and guidance in this investigation.**

For the chemotherapy of brain tumours, administration techniques of cytostatic agents are usually divided into three routes: systemic, intra-arterial, and intrathecal or intratumoral administration.

Intrathecal administration, however, is not so frequently used, for this route of application may subject the normal brain tissue to the action of the drug and be liable to cause severe neurological dysfunction (Heppner, Diemath and Jenkner, 1961; Heppner and Diemath, 1963; Hockey and Mealey, 1965; Franco and Mealey, 1967).

The purpose of the present paper is to select cytostatic agents for intrathecal chemotherapy.

### MATERIALS AND METHODS

Experiments were conducted under the following headings:

I. *To select cytostatic agents for intrathecal administration from the viewpoint of toxic side effects.*

(a) Destruction of the blood–brain barrier—brain oedema—which can be presumed from measuring the uptake ratio by brain tissue of RISA (radio-iodinated serum albumin) injected intravenously, was studied after intracisternal administrations of cytostatic agents in rabbits.

(b) Changes of body weight were checked following intrathecal administration of cytostatic agents in rats.

II. *To evaluate the effect of intrathecal administration of cytostatic agents on survival period of intracranially tumour transplanted rats.*

I. (a) Four rabbits weighing 1·8 to 2·2 kg. in each group were employed in this study. Each animal was anaesthetized lightly with sodium pentobarbital, cisternal puncture was accomplished using a 25-gauge needle and immediately afterwards 0·5 ml. of cerebrospinal fluid per kg. of body weight was withdrawn slowly, and various doses of cytostatic agent were injected intracisternally. The cytostatic agent was dissolved in the same volume of sterile water as that of cerebrospinal fluid withdrawn and was prepared by the addition of sodium chloride to isotonic solution. At 24 hours later RISA 10 $\mu$Ci. per kg., which hardly penetrates the normal blood–brain barrier and produces no alterations in the vessels of the brain, was given intravenously. At 48 hours after administration of the cytostatic agent, intravenous sodium pentobarbital was used as anaesthetic agent and left thoracotomy was performed. A sample of blood serum was collected by cardiac puncture for counting radioactivity and the animal was exsanguinated by an incision in the right auricle. Through a catheter inserted into the aorta, the brain and upper body were perfused with 200 ml. of saline. The entire brain was then removed, rinsed with saline immediately and blotted with reasonable firmness on a piece of filter paper. After the pia mater had been removed, approximately 1 g. each of brain tissue was excised from the right parietal lobe and from the cerebellum, was pushed to the bottom of a test tube, and weighed accurately. Radioactivities of the brain tissues and sera were counted in a scintillation counter. Uptake ratio by brain tissue of RISA was calculated by the following formula:

$$\frac{\text{Counts/min./g. of brain tissue}}{\text{Counts/min./ml. of serum}} \times 10^4$$

I. (b) Five rats weighing approximately 100 g. in each group were employed in this experiment. The animals were anaesthetized with ether and the cytostatic agent was injected intrathecally. The technique employed has been described by Lindberg and Ernster (1950). The cytostatic agent was dissolved in 0·1 ml. of water per 100 g. of body weight and prepared by addition of sodium chloride to isotonic solution. Food and water were supplied *ad libitum*, and the animals were weighed every other day after administrations of drugs.

The cytostatic agents investigated in these experiments were: Endoxan (cyclophosphamide), mitomycin C, Nitromin (nitrogen mustard N-oxide), Thio-TEPA (triethylene thiophosphoramide), Methotrexate (amethopterin), 5-fluorouracil and Vincristine. As a control, isotonic saline solution was used.

II. Ten rats, weighing approximately 100 g., in each group were used in this experiment. Two to three $\times$ $10^5$ cells of Yoshida ascites sarcoma were inoculated intracranially by the method of Lindberg and Ernster (1950). Two days after the tumour transplantation, cytostatic agent was administered intrathecally or intraperitoneally once only or once a day for 3 days, and the survival times of the rats were observed.

### RESULTS

I. (a) The results obtained in this experiment are shown in Fig. 1. The groups which showed little difference in RISA uptake from the control group were the following groups: Endoxan (15 mg./kg.), mitomycin C (0·03 mg./kg.), Thio-TEPA (0·1 mg./kg.) and Methotrexate (0·15, 0·3 mg./kg.). On the contrary, the groups: mitomycin C (0·06, 0·3 mg./kg.) and Vincristine (0·2 mg./kg.) showed

FIG. 1.—Relative specific activities of brain to serum levels of $I^{131}$ (RISA) in rabbits killed 48 hours after intracisternal administration of Endoxan (EX), mitomycin C (MMC), Nitromin ($HN_2$-O), Thio-TEPA, Methotrexate (MTX), 5-Fluorouracil (5-FU), or Vincristine (VCR).

significantly higher RISA uptake than those of the control. In the group: Methotrexate (1 mg./kg.), there was slightly increased RISA uptake especially in cerebrum. The animals in the group: Nitromin (0·8 mg./kg.) died within 24 hours, and the majority in the group: 5-fluorouracil (2·5 mg./kg.), within 48 hours after administration of the drug.

I. (b) The results obtained are given in Fig. 2, 3 and 4. The animals in the groups: Endoxan (15 mg./kg.), mitomycin C (0·06 mg./kg.) and Methotrexate (0·15, 0·3 mg./kg.) gained weight day by day as well as the control group and also in the group: Thio-TEPA (0·1 mg./kg.), although there were some differences from the controls, gained weight favourably. However, the growth of animals in the other groups was inhibited significantly following administration of the cytostatic agent and some of the animals died after marked decrease of body weight.

II. The average life span of each group is shown in Fig. 5. The survival time of the control group was 6·1 $\pm$ 0·4 days. In the groups: Endoxan and Methotrexate (except the group: 5·0 mg./kg. for 3 days), the larger the quantity of the drug administered, either intraperitoneally or intrathecally, the longer was the life span of the animal observed. Furthermore, the life span of the group in which the drug was administered intrathecally was remarkably longer than that of the group in which the same quantity of the drug was administered intraperitoneally. As to the group in which mitomycin C was administered intrathecally, the survival time of the group in which the quantity of the drug administered

**FIG. 2.—Changes of body weight following intrathecal administration of Endoxan (EX) or mitomycin C (MMC) in rats.**

**FIG. 3.—Changes of body weight following intrathecal administration of Nitromin ($HN_2$-O) or thio-TEPA in rats.**

FIG. 4.—Changes of body weight following intrathecal administration of Methotrexate (MTX), 5-fluorouracil (5-FU) or Vincristine in rats.

was more than 0·06 mg./kg. was rather shorter as compared with that of the group in which the quantity of the drug administered was 0·03 mg./kg. for 3 days. And in the group: mitomycin C (0·03 mg./kg. for 3 days), the life span of the intrathecally administered drug group was significantly longer than that of the intraperitoneally administered drug group.

### DISCUSSION

In the chemotherapy of brain tumours, the blood–brain barrier limits the passage of the cytostatic agent from blood to brain and by so doing, protects normal brain tissue from the neurotoxicity of the drug (Rall, 1965). Cytostatic agents administered intrathecally are however absorbed directly into the brain and are liable to injure normal brain tissue. Therefore, administration by this route must be exploited by careful selection of drugs and their doses, and as yet little work has been done to study this.

Harbauer *et al.* (1965) investigated the electroencephalogram of rabbits following the intracranial extracerebral application of different cytostatic agents—Endoxan, Nitromin, Trenimon (trisethyleneimino-benzochinon) and tetraethyleneiminobenzochinon—and suggested that only Endoxan seemed to be suitable for local intracranial application. Extensive studies on such application have been conducted by Heppner and his associates (Heppner *et al.*, 1961; Heppner and Diemath, 1963). For the first time, they tested the use of various cytostatic agents locally on the guinea-pig brain. Then, by means of staining reactions, spectrographical methods and radioactive tracers, they investigated the behaviour of cytostatic agents contained in pieces of gelatine sponge on the brain and proved

FIG. 5.—Effects of intrathecal or intraperitoneal administration of Endoxan (EX), mitomycin C (MMC) or Methotrexate (MTX) on survival time of intracranially Yoshida sarcoma-transplanted rats.

that Endoxan is the best agent for such application. Furthermore, this technique has been applied, by him and his co-workers (Heppner, 1963), clinically to patients suffering from malignant brain tumours.

In our experiments we have tried to study, by different means from those described in the earlier reports, the selection of cytostatic agents for intrathecal administration, and the data obtained indicate that Endoxan, Methotrexate, and lower dose of mitomycin C may be useful for intrathecal chemotherapy of brain tumours.

In the respect that Endoxan has little toxicity to the central nervous system when administered intrathecally as compared with other alkylating agents, our results are in remarkable agreement with earlier workers (Heppner, Diemath and Jenkner, 1961; Heppner and Diemath, 1963; Harbauer *et al.*, 1965). A possible explanation for the results could be discussed in relation to the concept that Endoxan is a latent alkylating agent, which is almost inactive *in vitro* and becomes biologically active *in vivo* only upon appropriate activation (Arnold, Bourseaux and Brock, 1958; Brock, 1963). If this conception is valid and also the activation of Endoxan takes place neither in the normal brain nor in cerebrospinal fluid, the findings that Endoxan has little toxicity on the local normal brain tissue by intrathecal administration could be explained. Since the demonstration by Foley *et al.* (1961), the view has been widely held that Endoxan is activated primarily by the liver and not by tumour, though at first it was believed to be activated in tumour cells by enzymic hydrolysis. Chirigos *et al.* (1962) demonstrated that Endoxan had no effect on intracerebrally transplanted L-1210 tumour in the mouse, either by intracerebral or by intraperitoneal administration. In this experiment, however, intrathecal administration of Endoxan extended the survival time of intracranially Yoshida ascites sarcoma transplanted rats. Differences of opinion concerning the cytostatic effect of intracranial administration of Endoxan seem to justify further examination.

There is a photometrical method of analysis of alkylating agents described by Epstein *et al.* (1955) and by Friedman and Boger (1961). Recently, Morita *et al.* (1965) have extended its application to analyses of Endoxan, and the unmetabolized inactive form of Endoxan, which has no alkylating activity, was measured as well as the active metabolites, which have alkylating activity. Yamada *et al.* (1968) have investigated the distribution of Endoxan in cerebrospinal fluid after intracisternal administration by this analytical method in dogs and demonstrated that the concentration of the substances in alkylating state in cerebrospinal fluid reached a considerably high level for a few hours after intracisternal administration of Endoxan, though the concentration ratio of active to inactive substances of Endoxan in cerebrospinal fluid was steadily very low. It was suggested that biochemical activation of Endoxan would not take place in the cerebrospinal fluid, but cerebrospinal fluid after intrathecal administration of Endoxan contains considerable quantities of active substance which would chemically come to be in alkylating state, for the concentration of Endoxan is maintained at an extremely high level in the cerebrospinal fluid. The result of this experiment suggests that intrathecal administration of Endoxan may be useful for the chemotherapy of brain tumours.

Mitomycin C is a kind of antibiotic and it has been demonstrated that the proliferation of HeLa cells which had been treated with mitomycin C at a concentration of 1 $\mu$g./ml. for 1 hour was almost completely inhibited (Doi *et al.*, 1967). Intrathecal administration of mitomycin C (0·03 mg./kg. for 3 days) resulted in a significantly longer extension of survival time of intracranially tumour transplanted rats as compared with the intraperitoneal administration of the same quantity of the drug, but it also appeared obvious that the larger dose of mitomycin C by intrathecal administration was liable to cause severe brain damage and rather to shorten the survival time of intracranial tumour-bearing rats. These observations indicate that intrathecal administration of mitomycin C may be useful for chemotherapy of brain tumours, but should be tried with great caution.

Methotrexate is a kind of folic acid antagonist and should prove most effective in tumours of the central nervous system where DNA turnover is very low as compared to tumour (Hevesy and Ottesen, 1943). Since the demonstration of Sansone (1954) that intrathecal administration of Methotrexate was useful for the treatment of meningeal leukaemia, the application of the drug has been widely used (Whiteside *et al.*, 1958; Rieselbach *et al.*, 1963; Hyman *et al.*, 1965). Rubin *et al.* (1966) have extended the intrathecal application of the drug to cerebrospinal fluid perfusion for the treatment of brain tumour, and recently Norrell and Wilson (1967) have developed a method of repetitive intrathecal administration of the drug with the use of components of a ventriculo-atrial shunt.

Wollner *et al.* (1959) described local and systemic toxicity following intracisternal administration of Methotrexate in dogs, and Rall *et al.* (1962) reported that Methotrexate was epileptogenic in unanaesthetized dogs when given intrathecally in concentration greater than 1 mg./ml. Rubin *et al.* (1966) found that 0·5–1·0 mg./ml. of Methotrexate in cerebrospinal fluid was tolerated for cerebrospinal fluid perfusion, and Norrell and Wilson (1967) administered Methotrexate (0·25–0·375 mg./kg.; concentration: 5 mg./ml.) through the reservoir into the cerebrospinal fluid space clinically.

There is remarkable agreement between the earlier works and our findings, and the available evidence indicates that Methotrexate is one of the most suitable drugs for the intrathecal chemotherapy of brain tumours.

Based on the experimental findings in this series, 27 patients diagnosed as having malignant brain tumours have been treated with intrathecal chemotherapy in our clinic. One mg. of mitomycin C or 100 mg. of Endoxan in 20 ml. of physiological saline solution was spread on the tumour resected area at operation, and 5 to 10 mg. of Methotrexate, twice a week was injected into the tumour resected brain cavity through an Ommaya's reservoir (Ratcheson and Ommaya, 1968) in the post-operative period. No serious complications followed the application of the drug, and, though a final conclusion as to the therapeutic effects cannot yet be drawn from the clinical data because of the small number of cases and the shortness of the follow-up period, we have a definite impression that the fate of those who were treated with intrathecal chemotherapy in addition to surgery was somewhat better than those who were treated only with surgery.

This study was supported in part by a grant in aid for Fundamental Scientific Research from the Japan Welfare Ministry.

### REFERENCES

Arnold, H., Bourseaux, F. and Brock, N.—(1958) *Naturwissenschaften*, **45**, 64.

Brock, N.—(1963) 2nd International Symposium of Chemotherapy, Naples 1961, Part 3, 1.

Chirigos, M. A., Humphreys, S. R. and Goldin, A.—(1962) *Cancer Res.*, **22**, 187.

Doi, O., Aoki, Y., Kosaki, G., Takai, S. and Higashi, H.—(1967) *Gann*, **58**, 125.

Epstein, J., Rosenthal, R. W. and Ess, R. J.—(1955) *Analyt. Chem.*, **27**, 1955.

Foley, G. E., Friedman, O. M. and Drolet, B. P.—(1961) *Cancer Res.*, **21**, 67.

Franco, J. M. and Mealey, J., Jr.—(1967) *Surg. Forum*, **18**, 456.

Friedman, O. M. and Boger, E.—(1961) *Analyt. Chem.*, **33**, 906.

Harbauer, G., Herrmann, H. D., Loew, F. and Schmidt, A.—(1965) *Acta neurochir.*, **12**, 554.

Heppner, F.—(1963) *Excerpta med.*, International Congress Series No. 60, 2nd European Congress of Neurological Surgery, 71.

Heppner, F. and Diemath, D. E.—(1963) *Acta neurochir.*, **11**, 287.

Heppner, F., Diemath, H. E. and Jenker, F. L.—(1961) *Wien. med. Wschr.*, **111**, 725.

Hevesy, G. and Ottesen, J.—(1943) *Acta physiol. scand.*, **5**, 237.

Hockey, A. A. and Mealey, J., Jr.—(1965) *Surg. Forum*, **16** 427.

Hyman, C. B., Bogle, J. M., Brubaker, C. A., Williams, K. and Hammond, D.—(1965) *Blood*, **25**, 13.

Lindberg, O. and Ernster, L.—(1950) *Biochem. J.*, **46**, 43.

Morita, M., Iwata, I., Tochino, Y. and Mineshita, T.—(1965) *Proc. Jap. Cancer Ass. gen. Mtg.*, **24**, 320.

Norrell, H. and Wilson, C.—(1967) *J. Am. med. Ass.*, **201**, 93.

Rall, D. P.—(1965) *Cancer Res.*, **25**, 1572.

Rall, D. P., Rieselbach, R. E., Oliverio, V. T. and Morse, E.—(1962) *Cancer Chemother. Rep.*, **16**, 187.

RATCHESON, R. A. AND OMMAYA, A. K.—(1968) *New Engl. J. Med.*, **279**, 1025.
RIESELBACH, R. E., MORSE, E. E., RALL, D. P., FREI, E. III, AND FREIREICH, E. J.—(1963) *Archs intern. Med.*, **3**, 620.
RUBIN, R. C., OMMAYA, A. K., HENDERSON, E. S., BERING, E. A. AND RALL, D. P.—(1966) *Neurology, Minneap.*, **16**, 680.
SANSONE, G.—(1954) *Annls paediat.*, **183**, 33.
WHITESIDE, J. A., PHILIPS, F. S., DARGEON, H. W. AND BURCHENAL, J. H.—(1958) *Archs intern. Med.*, **101**, 279.
WOLLNER, N., MURPHY, M. L. AND GORDON, C. S.—(1959) Abstract, *Proc. Am. Ass. Cancer Res.*, **3**, 74.
YAMADA, R., KANAI, N., KURODA, R., HAYAKAWA, T., HIGASHI, H., MOGAMI, H. AND JINNAI, D.—(1968) *Med. J. Osaka Univ.*, **18**, 373.